# Peripartum cardiomyopathy: a review

**DOI:** 10.1007/s10741-020-10061-x

**Published:** 2021-06-17

**Authors:** Corina Iorgoveanu, Ahmed Zaghloul, Mahi Ashwath

**Affiliations:** Division of Cardiovascular Medicine, University of Iowa Hospitals and Clinics, University of Iowa Health Care, 200 Hawkins Drive, Iowa City, IA 52242 USA

**Keywords:** Peripartum cardiomyopathy, Heart failure

## Abstract

Peripartum cardiomyopathy is a form of idiopathic systolic heart failure which occurs during the end of pregnancy or the early post-partum in the absence of an identifiable etiology. The exact pathogenesis remains unknown, and the incidence is higher in African ancestry, multiparous and hypertensive women, or older maternal age. Delay in diagnosis is common, mainly because symptoms of heart failure mimic those of normal pregnancy. Echocardiography showing decreased myocardial function is at the center of the diagnosis. Management relies on the general guidelines of management of other forms of non-ischemic cardiomyopathy; however, special attention should be paid when choosing medications to ensure fetal safety. Outcomes can be variable and can range from complete recovery to persistent heart failure requiring transplant or even death. High rates of relapse with subsequent pregnancies can occur, especially with incomplete myocardial recovery. Additional research about the etiology, experimental drugs, prognosis, and duration of treatment after recovery are needed.

## Introduction

Peripartum cardiomyopathy (PPCM) is a form of systolic heart failure (HF) with reduced ejection fraction that occurs in women during last trimester of pregnancy or in the first 5 to 6 months of the postpartum period. Initially described in 1849 [1], it was recognized as a distinct clinical entity in the 1930s [2]. Diagnosis of PPCM is challenging since symptoms may mimic those encountered in a normal pregnancy. Because of this, delays in diagnosis and subsequent complications may occur. This clinical review will define the disease and describe the pathophysiology, management, prognostic factors, and counseling considerations.

## Definition

PPCM is a diagnosis of exclusion in women presenting with heart failure and systolic dysfunction of unclear identifiable etiology. The diagnosis is based on three criteria based on the 2010 ESC Working Group Definition including development of heart failure (HF) toward the end of pregnancy or in the months following delivery, absence of another identifiable cause of HF, and left ventricular (LV) systolic dysfunction with an LV ejection fraction (LVEF) generally < 45 percent [3]. The definition has since been expanded to include the last trimester of pregnancy and the first 6 months postpartum.

## Epidemiology

The incidence of PPCM varies widely, depending mostly on racial and geographical background of the patients [4]. Overall, multiple studies have shown that Africans and African Americans seem to be at a higher risk for developing PPCM, with estimates of 1:100 pregnancies [3, 5]. In one large United States (US) cohort study of well-phenotyped patients, it was found that African American women were not only at a higher risk of developing a more severe form of PPCM but they were also diagnosed with PPCM at a younger age and later in the post-partum period [6].

A cross-sectional study using more than 14 million hospitalizations for pregnancy in the US between 2004 and 2006 showed that the rate of hospitalizations for PPCM was 0.18 per 1000 deliveries and 0.28 for other cardiomyopathies [7].

A recent study using the US Nationwide Inpatient Sample databases found that the incidence of PPCM increased from one in 1181 live births to one in 849 live births from 2004 to 2011. Proposed explanations are increased rates of advanced maternal age, pre-eclampsia, multiple gestations, and rising prevalence of cardiovascular risk factors [8].

## Pathophysiology

Although a complete understanding of the etiology of PPCM is still ill-defined, many potential causes have been suggested. These include genetic factors, myocarditis (viral or antigen induced), pathological immune response to pregnancy (i.e., to fetal antigens), pathological response to hemodynamic changes of pregnancy (i.e., increased cardiac demand of pregnancy, hypertension), hormonal abnormalities, angiogenic imbalance, stress activated cytokines, and nutritional deficiencies (e.g., selenium) [9–11].

## Risk factors

Although no casual association has been proven, a higher incidence of PPCM was seen in multiparous women, African ancestry, maternal age > 30 years, twin pregnancies, hypertensive women, women with history of eclampsia/preeclampsia, in vitro fertilization, or with use of tocolytic agents [10, 12].

## Genetic predisposition

Although PPCM has been classified as a non-genetic form of dilated cardiomyopathy, several studies have documented familial clustering [13–16], supporting the theory that PPCM may have a hereditary or genetic component. One of the first reflections on the familial clustering of PPCM was reported in a retrospective case series of 17 patients, among which 3 had a known family history of PPCM, then described as “post-natal heart failure” [17]. First systematized approaches to examining the genetics of PPCM as part of the spectrum of Dilated Cardiomyopathy (DCM) were studies done by Morales et al. and van Spaendonck-Zwarts et al. [18, 19]. Their results promote the theory that pregnancy-associated changes may trigger dormant disease susceptibility into active PPCM. Recent research was conducted to sequence DNA from 172 women with diagnosis of PPCM in order to establish the contribution of variants in 43 genes that have been previously associated with DCM [20]. The prevalence of different variant types was compared between patients with DCM and population controls. It was noted that the prevalence of the truncating variants (26 of 172: 15%) was remarkably higher than that in a reference population (4.7%), but was comparable with that in a group of patients with DCM (17%). Two thirds of identified truncating variants were TTN, the gene encoding the protein titin, which is the third most abundant striated-muscle protein. Titin accounts for 1 of the 3 major filaments of the cardiac sarcomere, acting as the essential scaffold for the development of the sarcomere, and it is also the defining structural component that spans from the Z-disk to the M-line. The authors also distinguished heterozygous truncations in 2 genes on the X chromosome (DMD and LAMP2), as well as truncations in DSP, MYH6, SYNM, TPM1, and VCL. In a subgroup of 83 women, prospectively followed in the Investigations in Pregnancy Associated Cardiomyopathy ( IPAC) study, the presence of TTN truncating variants was particularly correlated with a lower LVEF at 1-year follow-up [20]. Herman et al. conducted a study which showed that TTN truncating mutations were a common cause of DCM, occurring in 25% of familial cases and in 18% of sporadic cases [21].

Although these studies bring up new awareness into potential genetic mechanisms of PPCM, evidence of causation varied, leading to a limited genetic evidence. Therefore, a comprehensive family history is essential in patients with PPCM, but routine genetic testing is not routinely indicated except in those cases with a history of cardiomyopathy.

## Oxidative stress and prolactin

Oxidative stress seems to be part of the key factors involved in disease pathogenesis. Serum markers of inflammation and apoptosis are significantly elevated, concordant with the proposition that oxidative stress may contribute to the pathogenesis of PPCM [4].

In normal pregnancy, in order to build up the maternal defense mechanism against pathogens, given higher infection risk than normal, the production of reactive oxygen species (ROS) increases, which culminates in the last trimester. As an effort to counterbalance the deleterious effect of ROS, the antioxidant capacity also increases during normal pregnancy [22, 23]. Unfortunately, unlike normal pregnancy, in PPCM the level of oxidative stress is more amplified and it does not match with an increase in anti-oxidant levels; thus, eliciting a shift towards increased oxidative stress, which is thought to predispose to PPCM [23].

Kleiner et al. proposed an explanation for the increased oxidative stress in PPCM based on a genetic mouse model with cardiomyocyte restricted deletion of signal transducer and activator of transcription 3 (STAT-3) [23]. The activation of STAT3 generates a defensive effect on hypoxia/reoxygenation-induced cardiomyocyte injury, mainly by upregulating the ROS scavenging enzyme manganese superoxide dismutase MnSOD [13]. It was noted that the female mice with a homozygous or heterozygous cardiomyocyte-specific knockout of STAT3 develop PPCM in a dose-dependent manner. Oxidative stress triggers, through unclear mechanisms, cardiomyocytes to express cathepsin D, which in turn, enhances the conversion of prolactin into an antiangiogenic and proapoptotic 16 kDa derivative. The production of 16 kDa significantly promotes cardiac injury caused by oxidative stress, further reinforced by injection of adenoviral vectors expressing 16 kDa in non-pregnant mice, which led to cardiac dysfunction and decreased myocardial capillary density [23–25].

This theory was further reinforced by the beneficial effect of blockade of prolactin cleavage with bromocriptine in some limited studies [4, 23, 26, 27] and by the elevated levels of the oxidized low-density lipoprotein (oxo-LDL), which is a marker of oxidative stress [23].

## Inflammation

A pro-inflammatory state might play a role in the pathophysiology of PPCM. The theory was supported by studies that found significant circulating levels of pro-inflammatory cytokines (interleukin-6, tumor necrosis factor-α, interferon-ϒ, and C-reactive protein, soluble death receptor sFas/Apo1). This process is further emphasized by the clinical benefit of the antiinflammatory agent pentoxifylline in a nonrandomized trial in 58 patients with PPCM [28].

## Myocarditis (viral myocarditis and antigen-induced autoimmune myocarditis)

### Viral myocarditis

Results from multicenter IPAC study showed that patients with PPCM had decreased levels of natural killer cells [29], which are involved in destruction of virus-infected cells. This has led to the hypothesis that a viral cardiomyopathy might be another possible cause of PPCM. This was first reported by Goulet et al. [30]. The first study to identify viral genomes in cardiac tissue of PPCM patients was done by Bultmann et al. in 2005. The viruses identified in 8 out of 26 patients included Epstein Barr virus, human cytomegalovirus, human herpes virus 6, and parvovirus B19 [14].

On the other hand, Lamparter et al. found no evidence of viral infection in endomyocardial biopsy samples from 7 PPCM patients. Their patients underwent PCR testing for adenovirus, coxsackievirus B, influenza virus A, cytomegalovirus, Epstein Barr virus, herpes simplex virus 1 + 2, human herpes virus 6, and parvovirus B19 [15]. It is unclear if viruses play any role in the pathogenesis at this time.

### Antigen-induced autoimmune myocarditis

Several research studies supported the theory that because of natural immunosuppression during pregnancy, fetal cells are not rejected when they escape into the maternal circulation and induce an autoimmune myocarditis. This process is known as microchimerism, when two genetically different populations of cells emerge in the same tissue or organ. Kara et al. conducted an experimental study that demonstrated that fetal cells selectively concentrate on sites of maternal cardiac injury, where they eventually differentiate into diverse cardiac cell types. Although those fetal cells were found to express a variety of markers, the novel findings in this study was that approximately 40% of these cells expressed Caudal-related homeobox2 (Cdx2). Cdx2 is expressed in trophoblast stem cells but it is absent in the mature trophoblast. This led to the suggestion that the cells with regenerative potential may have originated from the placenta [31].

### Malnutrition:

Several studies mentioned that deficiency in selenium and other micronutrients might play a role in the pathogenesis of PPCM [32–34]. This theory was not supported by Fett et al. who found that neither selenium deficiency nor low serum levels of other micronutrients (vitamin A, B12, C,E) play a notable role in the etiology of PPCM in Haitian women [35].

## Diagnosis

### Clinical presentation

Most women with pre-existing cardiac disease develop symptomatic HF in the 2^nd^ trimester, when the maximal cardiovascular changes occur. In contrast, the majority of women with PPCM typically develop symptoms in the first month postpartum, but it can occur in the third trimester or up to 6 months postpartum [36]. While most patients present with typical heart failure signs and symptoms, patients can also present with thromboembolic complications, life-threatening arrhythmias, and even cardiac death [16].

Common symptomatology includes congestive symptoms (dyspnea on exertion, orthopnea, paroxysmal nocturnal dyspnea, dry cough, or pedal edema) or nonspecific symptoms, such as fatigue, malaise, palpitations, lightheadedness, or abdominal discomfort [10]. Diagnosis of PPCM is challenging since symptoms may mimic those encountered in a normal pregnancy. When symptoms persist or are disproportionate to what is expected for pregnancy, PPCM should be suspected and evaluation undertaken.

Physical examination often reveals jugular venous distention, displaced apical impulse, presence of S3, pansystolic murmur consistent with functional mitral regurgitation, pulmonary rales, or peripheral edema. In rarer instances, patients can present with cardiogenic shock, severe arrhythmias, or neurological deficits secondary to cardiac thrombus embolization [36, 37].

A prospective study (ESC-EORP registry) which included 740 women with suspected peripartum cardiomyopathy showed that traditional signs were unreliable when seeking to recognize PPCM, with 42% of patients having no signs of peripheral edema and 41% with no pulmonary rales. A quarter of patients with mild symptoms (NYHA I/II) had a LVEF < 25%, suggesting poor relationship between symptoms and degree of myocardial dysfunction. Thus, a high index of suspicion and quick approach for cardiac testing are required. A quarter of patients had concomitant preeclampsia, which has been presumed to share pathophysiological mechanisms with PPCM. Deep venous thrombosis, pulmonary embolism, and ischemic stroke were diagnosed in approximatively 7% of patients at 6 months from diagnosis. In this registry, mortality at 6 months was 6%, and it was mainly due to heart failure (42%), and sudden death (30%) [38].

Some studies suggest that patients who present with heart failure symptomatology before delivery might have a worse outcome, including decreased rate of left ventricular function recovery [39]. *Diagnostic testing:* Since PPCM is a diagnosis of exclusion, a thorough investigation is required in order to rule out other causes of cardiomyopathy. The diagnosis can be challenging because of the potential impediment in initially differentiating PPCM from worsening of a pre-existing and unrecognized heart disease by pregnancy-mediated hemodynamic changes.

Unfortunately, no specific diagnostic test for PPCM is available at present. The current diagnostic criteria for PPCM include development of an unclear cause of HF in last month of pregnancy up until 6 months postpartum in the absence of known preexisting heart disease, with an LVEF of < 45% and often (but not required) LV dilatation [10]. Echocardiography should be performed in any suspected case and remains at the central point of diagnosis.

Initial blood profiling is aimed at excluding other causes that can explain patient’s symptomatology (significant anemia, active infection, thyroid dysfunction, electrolyte abnormalities, renal or liver dysfunction) [36]. Initial blood works also assist with prognosis, as it was found that BNP and NT-proBNP levels correlate with clinical outcomes, including recovery of LVEF [4].

Electrocardiographic findings may be nonspecific, but a normal electrocardiogram does not rule out PPCM [37]. Sinus tachycardia and arrhythmia, atrial fibrillation/flutter, and ventricular tachycardia have been reported [40]. Despite the nonspecific findings, QRS prolongation of more than 120 ms is related to increased mortality [41]. Chest radiography may show cardiomegaly or signs of congestion (vascular redistribution, pleural effusions, interstitial edema) [36]. Echocardiographic evaluation of the LVEF is key in evaluation of PPCM. Echocardiogram does not only confirm the diagnosis but also evaluates for other causes of heart failure (valvular disorder or any other structural abnormalities), assesses for complications of PPCM (e.g., LV thrombus), and it also provides prognostic data [3, 41].

Although echocardiographic finding of LVEF less than 45% is necessary to make the diagnosis of PPCM, other findings can also be present, including left ventricular dilatation, four-chamber enlargement, mitral or tricuspid regurgitation, elevated pulmonary artery pressures, and right ventricular enlargement [40]. A left-ventricular end-systolic diameter of < 5.5 cm portends better cure rates and shorter recovery times [10, 40]. Intracardiac thrombus has been visualized on initial echocardiogram in about 10 to 17% of patients with PPCM, posing an increased incidence of thromboembolism [40]. Conventionally used in postpartum period due to fetal toxicity of gadolinium, cardiac magnetic resonance imaging may provide a more precise measurement of chamber volumes and ventricular function than echocardiogram [40].

Since there is no histological criteria to confirm PPCM, endomyocardial biopsy is not typically proposed, but can be considered if there is suspicion for an alternative diagnosis, especially giant cell myocarditis, which would require a different treatment strategy [25], especially in the setting of significant cardiomyopathy, leading to cardiogenic shock.

### Differential diagnosis

PPCM is a diagnosis of exclusion. Due to the pregnancy associated hemodynamic changes, some pre- existing cardiac lesions may become manifest during pregnancy. It is important to consider pre-existing cardiomyopathy that can be unmasked during pregnancy. Most of these patients present in the second trimester, if they are not diagnosed antepartum, in contrast to PPCM patients who present in the early postpartum, although the timing can overlap.

Pre-existing acquired or congenital valvular heart disease including mitral or aortic stenosis or regurgitation can unmasked by pregnancy and should be excluded with an echo. Pre-existing undetected congenital heart disease lesions including atrial septal defects, ventricular septal defects, and patent ductus arteriosus can sometimes be unmasked during pregnancy. The clinical presentation and echocardiography are helpful in distinguishing these lesions.

Hypertensive heart disease should be considered in cases of significant longstanding hypertension. Hypertrophic cardiomyopathy and noncompaction cardiomyopathy are in the differential and can be excluded by echo or cardiac MRI.

Myocarditis should be considered if there is a viral prodrome or fulminant presentation.

Ischemia due to thromboembolic disease or coronary artery dissection can be excluded based on the presentation with anginal chest pain, EKG and cardiac biomarker changes, and regional wall motion abnormalities on echocardiogram.

### Management

A multidisciplinary approach involving cardiologist, obstetrician, intensivist, and pediatrician is crucial for management of PPCM patients [3].

Initial management relies on the general guidelines of other forms of nonischemic cardiomyopathy, but a special attention is required when choosing agents that have a safe profile in the settings of pregnancy and lactation [36].

### During pregnancy

Management is based focus on relief of symptoms, acute, and chronic therapies (Table 1).

**Table 1:** Clinical scenario, treatment, and level of care during pregnancy

	Clinical scenario	Treatment	Level of care
Mild PPMC	• Subacute HF • Hemodynamic stability	• Oral HF medications • Oral diuretics	• General ward • Ambulatory treatment in selected cases
Moderate PPMC	• Acute HF • Hemodynamic stability • Respiratory failure	• Oral HF medication • Diuretics i.v. • Consider bromocriptine and anticoagulation • Optimization of oxygenation	• Intermediate care
Serve PPMC	• Cardiogenic shock • Hemodynamic stability • Respiratory failure	• Diuretics i.v • Vasodilators i.v (nitroglycerin) • Inotropes/pressors as needed • Consider bromocriptine and anticoagulation • Optimization of oxygenation	• Cardiac intensive care unit

Diuretics like furosemide and hydrochlorothiazide, although considered safe during pregnancy, should be used carefully as they may alter perfusion of the placenta and create potential distress to the fetus [41]. Diuretics should be generally reserved for symptomatic relief of pulmonary or peripheral edema [5]. Vasodilators like hydralazine, amlodipine, and nitroglycerin are considered safe during pregnancy, but nitroprusside should be avoided due to concerns of cyanide toxicity [41].

Due to concern of beta-blockers action on uterine tone, beta-1 selective blockers (metoprolol, bisoprolol, carvedilol) are the preferred agents. Because most available data is on metoprolol and due to its shorter half-life, this agent is most commonly prescribed. Since beta-blockers may cause fetal bradycardia and hypoglycemia, frequent fetal monitoring is warranted [5].

Angiotensin-converting enzyme (ACE) inhibitors, angiotensin II receptor blockers (ARBs), mineralocorticoid receptor antagonists, and neprilysin inhibitors should be avoided during pregnancy. ACE inhibitors and ARBs associated with teratogenic effects (renal dysgenesis, calvarial and pulmonary hypoplasia, neonatal anuric renal failure). These medications should be withheld also in the pre-conception period [42].

Anticoagulation: Since both pregnancy and heart failure are hypercoagulable states, anticoagulation has been proposed in PPCM patients if LVEF is less than 35%, but is not recommended in general [4, 43]. Anticoagulation is recommended if Bromocriptine is used or if a documented left ventricular thrombus is present. Bromocriptine is associated with increased thromboembolic events but is however considered investigational at this time as noted below. Warfarin is the preferred anticoagulant if the dose is less than 5 mg. Unfractionated and low molecular heparin are alternate agents. Warfarin is the preferred agent in the second and third trimesters. Warfarin should be discontinued prior to planned vaginal delivery and transitioned to dose adjusted continuous infusion of unfractionated heparin to avoid risks of fetal intracranial hemorrhage. The direct oral anticoagulants are not considered safe during pregnancy [40, 42].

When mechanical circulatory support is indicated, devices such as intra-aortic balloon counter pulsation (IABP), venoarterial extracorporeal membrane oxygenation (ECMO), LV assist device (LVAD), and Impella devices can be used as needed.

Although crucial in preserving hemodynamic stability in patient with CS, a prolonged course of vasopressors and inotropes is associated with high morbidity and mortality. The hemodynamic support with Impella heart pump for management of women with peripartum cardiogenic shock was analyzed by Elkayam et al. in a study which included 15 patients with a mean LVEF of 14%. More than 85% of these patients survived to discharge, and 50% of them had improvement of their baseline heart function. A similar outcome was highlighted by Sieweke et al. in a study that included 5 PPCM patients with refractory CS who received intensive care treatment with mechanical circulatory support (with either Impella device or in combination with veno-arterial extracorporeal membrane oxygenation in the setting of biventricular failure) along with bromocriptine therapy. It was observed that early LV unloading with the Impella device was related to LV recovery, which was not noted in patients with delayed Impella placement. LV unloading using Impella devices can be favorably used as a bridge to either heart recovery, implantation of more durable devices like LVAD or heart transplantation [38, 54, 55].

Older studies found that transplantation was performed in up to one-third of women with PPCM. More recent data suggest that transplantation rates can be much lower from 4 to 23% of patients. However, women who have been transplanted for PPCM have worse outcomes compared with other cardiac transplant recipients with higher mortality, higher incidence of rejection, poorer graft survival, and higher retransplantation rates [42, 44].

Future therapies: Several treatment strategies (Bromocriptine, immunosuppressive agents, intravenous immune globulin (IVIG), Pentoxifylline) remain experimental.

Bromocriptine is the most studied agent amongst these (Table 2). Because of the acknowledgment of the potential detrimental role of prolactin, bromocriptine has been suggested as a novel treatment of PPCM. Bromocriptine inhibits prolactin secretion, thus preventing the 16 kDA N terminal prolactin fragment from formation. Since several studies showed inconsistent clinical benefit, there are no definite guidelines in the US regarding the use of bromocriptine in PPCM patients [45]. The 2018 European Society of Cardiology guidelines include a IIb recommendation for the use of bromocriptine.

**Table 2:** Study, outcomes, and total number of patients/patients receiving bromocriptine

Study	Total number of patients/patients receiving bromocriptine	Type of study	Outcomes
Sliwa et al. [46]	20/10	Prospective single-center, randomized	Patients treated with bromocriptine had greater improvement in LVEF at 6 months than the control group, and fewer experienced the composite endpoint compared with the control group (death, NYHA class III/IV, or LVEF < 35% at 6 months)
Yameogo et al. [47]	96/48	Prospective single-center, randomized	LVEF was similar at entry but higher in the bromocriptine group at 2 weeks and at 3, 6, and 12 months. Mortality at 6 months was higher in both groups but lower in the bromocriptine-treated women
Hilfiker-Kleiner et al. [48]	2 different regiments of bromocriptine (1 week in 27 patients vs. 8 weeks in 31 patients)	Randomized trial	The investigators postulated an association between bromocriptine and the favorable outcomes. The study was limited by the lack of a control group not receiving bromocriptine
Haghikia et al. [27]	115/64	Observational	Bromocriptine in addition to standard therapy was associated with higher rate of improvement in LVEF, but there was no significant difference in overall rates of recovery
REBIRTH (Randomized Evaluation of Bromo-criptine in Myocardial Recovery Therapy)	200	A randomized double-blind, placebo control study	Under evaluation

Due to the association with thromboembolic phenomenon, therapeutic anticoagulation is recommended in conjunction with bromocriptine. Bromocriptine also stops production of breast milk making breastfeeding impossible [43].

The Myocarditis Treatment Trial [49] did not show any benefit of immunosuppressive medications, and given the risks associated with these medications, they are currently not widely used.

Likewise, IMAC (Controlled trial of Intravenous Immune Globulin in Recent Onset Dilated Cardiomyopathy) [49] trial failed to show any significant improvement of IVIG in patients with recent onset cardiomyopathy; thus, IVIG is not routinely recommended in PPCM patients.

Pentoxifylline was studied in small trials that yielded positive results. In the study by Sliwa et al. which involved 59 patients, Pentoxifylline was an independent predictor of favorable outcome with better LVEF, NYHA class, and survival [28].

Given the small sample sizes of the above experimental drugs, the generalizability of these studies is unclear.

### Delivery

In women with PPCM with advanced heart failure, prompt delivery can be considered for maternal cardiovascular indications or hemodynamic instability. Planned cesarean delivery is preferred for women with advanced HF requiring inotropic therapy or mechanical circulatory support.

Early delivery is not required if the maternal and fetal conditions are stable and most pregnant patients can safely be delivered vaginally.

### Postpartum

For women with peripartum cardiomyopathy who have delivered and are not breastfeeding, acute and chronic HF should be managed using standard GDMT therapy. Given the benefits of breastfeeding, women who are clinically stable should not be discouraged from breastfeeding as long as it is compatible with their heart failure medications.

In breast feeding women, beta blockers, enalapril, and spironolactone are compatible with breast feeding. ARBs, Neprilysin inhibitors, and Ivabradine do not have enough information and should be avoided during pregnancy and lactation. Captopril and enalapril were found in clinically insignificant amounts in the breast milk and are deemed to be compatible with breast feeding according to the American Academy of Pediatrics [50].

In the ESC-EORP registry, 30% of demises at 6 months were due to sudden cardiac death, which might suggest that defibrillators might play an important role. Wearable defibrillators can be an acceptable option to prevent sudden cardiac death while either monitoring for LVEF recovery or for patients awaiting heart transplantation [38]. Thus, it is reasonable to wait for 6 months of optimal medical therapy when considering the timing of implantable defibrillators or Cardiac Resynchronization Therapy.

### Prognosis

No significant long-term studies of the natural history of patients with PPCM have been published, so long-term implications remain unclear. Many studies have tried to investigate the predictors for LV recovery in patients with PPCM. Increased LV end-diastolic diameters (LVEDD ≥ 6 cm), LVEF < 35% at time of diagnosis, right ventricular dysfunction, African ancestry, older age, later diagnosis, and elevated inflammatory markers predict adverse outcomes and a lower probability of full recovery [16, 51]. The Task Force on the Management of Cardiovascular Diseases during Pregnancy of the European Society of Cardiology discourages subsequent pregnancy when the LVEF has not returned to the values from before pregnancy. Continuation of guideline-directed medical therapy (GDMT) is recommended for the long term in these patients. Women with PPCM with persistent left ventricular (LV) dysfunction or LV ejection fraction (LVEF) ≤ 25% at diagnosis are at high risk of recurrent PPCM and should avoid future pregnancy.

Patients with PPCM with recovered LVEF have a better prognosis but can have a high rate of PPCM recurrence with future pregnancies [52]. There is also a risk of future HF and LV dysfunction even without pregnancy [53]. These patients must remain under regular cardiac care. Specific recommendations re-continuation of GDMT cannot be made at this time. If subsequent pregnancies are planned, echocardiography should be performed, and dobutamine stress testing may be helpful to determine the contractile reserve and further risk-stratify the potential for recurrence [43].

In summary, PPCM is a cardiomyopathy of unknown etiology occurring in the last trimester or the first 6 months postpartum. Multidisciplinary care, with a focus on maternal and fetal well-being, is needed for the appropriate management of these patients.

### Counseling of women with PPCM

Heart Failure Association PPCM Study Group recommends a regular, 6-month follow-up with echocardiography until LVEF recovers to > 50%. Although there are no guidelines yet on whether goal directed medical therapy can be stopped once LVEF recovers, some of the PPCM study group members advocate for life-long therapy based on the risk of HF and LV dysfunction even without subsequent pregnancies. If the decision is to wean the medical therapy, then cautious and frequent monitoring of patient’s clinical and cardiac performance should be conducted. All subsequent pregnancies are associated with a risk of worsening of cardiac function, and thus, it is imperative for the patients to be evaluated by a multi-disciplinary team. From obstetric perspective, fetal growth should be evaluated by ultrasound every 4 weeks starting from week 24. Early delivery can be undertaken if a decline in cardiac function is noted, with an optimal target of 37 weeks. Post-partum, women with recent PPCM, should be informed about the use of contraceptives [56].

## References

[1] Ritchie C (1849). Clinical contributions to the pathology, diagnosis, and treatment of certain chronic diseases of the heart. Edinb Med Surg J.

[2] Hull EHF (1937). Toxic postpartum heart disease. New Orleans Med Surg J.

[3] Bauersachs J, König T, van der Meer P (2019). Pathophysiology, diagnosis and management of peripartum cardiomyopathy: a position statement from the Heart Failure Association of the European Society of Cardiology Study Group on peripartum cardiomyopathy. Eur J Heart Fail.

[4] Sliwa K, Hilfiker-Kleiner D, Petrie MC (2010). Current state of knowledge on aetiology, diagnosis, management, and therapy of peripartum cardiomyopathy: a position statement from the Heart Failure Association of the European Society of Cardiology Working Group on peripartum cardiomyopathy. Eur J Heart Fail.

[5] Stergiopoulos K, Lima FV (2019). Peripartum cardiomyopathy-diagnosis, management, and long term implications. Trends Cardiovasc Med.

[6] Irizarry OC, Levine LD, Lewey J (2017). Comparison of clinical characteristics and outcomes of peripartum cardiomyopathy between African American and non-African American Women. JAMA Cardiol.

[7] Kuklina EV, Callaghan WM (2010). Cardiomyopathy and other myocardial disorders among hospitalizations for pregnancy in the United States: 2004–2006. Obstet Gynecol.

[8] Kolte D, Khera S, Aronow WS (2014). Temporal trends in incidence and outcomes of peripartum cardiomyopathy in the United States: a nationwide population-based study. J Am Heart Assoc.

[9] Pearson GD, Veille JC, Rahimtoola S (2000). Peripartum cardiomyopathy: National Heart, Lung, and Blood Institute and Office of Rare Diseases (National Institutes of Health) workshop recommendations and review. JAMA.

[10] Bhattacharyya A, Basra SS, Sen P, Kar B (2012). Peripartum cardiomyopathy: a review. Tex Heart Inst J.

[11] Anderson JL, Horne BD (2010). Birthing the genetics of peripartum cardiomyopathy. Circulation.

[12] Ricke-Hoch M, Pfeffer TJ, Hilfiker-Kleiner D (2020). Peripartum cardiomyopathy: basic mechanisms and hope for new therapies. Cardiovasc Res.

[13] Negoro S, Kunisada K, Fujio Y (2001). Activation of signal transducer and activator of transcription 3 protects cardiomyocytes from hypoxia/reoxygenation-induced oxidative stress through the upregulation of manganese superoxide dismutase. Circulation.

[14] Bültmann BD, Klingel K, Näbauer M, Wallwiener D, Kandolf R (2005). High prevalence of viral genomes and inflammation in peripartum cardiomyopathy. Am J Obstet Gynecol.

[15] Lamparter S, Pankuweit S, Maisch B (2007). Clinical and immunologic characteristics in peripartum cardiomyopathy. Int J Cardiol.

[16] Tak BT, Cay S, Pamukcu HE (2020). Prognostic nutritional index as a novel marker for prediction of prognosis in patients with peripartum cardiomyopathy. Medicine (Baltimore).

[17] Pierce JA, Price BO, Joyce JW (1963). Familial occurrence of postpartal heart failure. Arch Intern Med.

[18] Morales A, Painter T, Li R (2010). Rare variant mutations in pregnancy-associated or peripartum cardiomyopathy. Circulation.

[19] van Spaendonck-Zwarts KY, Posafalvi A, van den Berg MP (2014). Titin gene mutations are common in families with both peripartum cardiomyopathy and dilated cardiomyopathy. Eur Heart J.

[20] Ware JS, Li J, Mazaika E (2016). Shared Genetic Predisposition in Peripartum and Dilated Cardiomyopathies. N Engl J Med.

[21] Herman DS, Lam L, Taylor MR (2012). Truncations of titin causing dilated cardiomyopathy. N Engl J Med.

[22] Toescu V, Nuttall SL, Martin U, Kendall MJ, Dunne F (2002). Oxidative stress and normal pregnancy. Clin Endocrinol (Oxf).

[23] Hilfiker-Kleiner D, Sliwa K (2014). Pathophysiology and epidemiology of peripartum cardiomyopathy. Nat Rev Cardiol.

[24] Honigberg MC, Givertz MM (2019). Peripartum cardiomyopathy. BMJ.

[25] Kim MJ, Shin MS (2017). Practicalmanagementofperipartumcardiomyopathy. Korean J Intern Med.

[26] Ballo P, Betti I, Mangialavori G, Chiodi L, Rapisardi G, Zuppiroli A (2012). Peripartum cardiomyopathy presenting with predominant left ventricular diastolic dysfunction: efficacy of bromocriptine. Case Rep Med.

[27] Haghikia A, Podewski E, Libhaber E (2013). Phenotyping and outcome on contemporary management in a German cohort of patients with peripartum cardiomyopathy. Basic Res Cardiol.

[28] Sliwa K, Skudicky D, Candy G, Bergemann A, Hopley M, Sareli P (2002). The addition of pentoxifylline to conventional therapy improves outcome in patients with peripartum cardiomyopathy. Eur J Heart Fail.

[29] McTiernan CF, Morel P, Cooper LT (2018). Circulating T-cell subsets, monocytes, and natural killer cells in peripartum cardiomyopathy: results from the multicenter IPAC study. J Card Fail.

[30] Goulet B, McMillan T, Bellet S (1937). Idiopathic myocardial degeneration associated with pregnancy and especially the puerpernium. Am J Med Sci.

[31] Kara RJ, Bolli P, Karakikes I (2012). Fetal cells traffic to injured maternal myocardium and undergo cardiac differentiation. Circ Res.

[32] Demakis JG, Rahimtoola SH, Sutton GC (1971). Naturalcourseofperipartumcardiomyopathy. Circulation.

[33] Brar SS, Khan SS, Sandhu GK (2007). Incidence, mortality, and racial differences in peripartum cardiomyopathy. Am J Cardiol.

[34] Cénac A, Simonoff M, Moretto P, Djibo A (1992). Alowplasmaseleniumisariskfactorforperipartum cardiomyopathy A comparative study in Sahelian Africa. Int J Cardiol.

[35] Fett JD, Sundstrom BJ, Etta King M, Ansari AA (2002). Mother-daughter peripartum cardiomyopathy. Int J Cardiol.

[36] Patel PA, Roy A, Javid R, Dalton JA (2017). A contemporary review of peripartum cardiomyopathy. Clin Med (Lond).

[37] Davis MB, Arany Z, McNamara DM, Goland S, Elkayam U (2020). Peripartum Cardiomyopathy: JACC state-of-the-art review. J Am Coll Cardiol.

[38] Sliwa K, Petrie MC, van der Meer P, et al. (2020) Clinical presentation, management, and 6-month outcomes in women with peripartum cardiomyopathy: an ESC EORP registry Eur Heart J 41(39):3787–3797 (In eng) 10.1093/eurheartj/ehaa455 PMC7846090 32840318

[39] Safirstein JG, Ro AS, Grandhi S, Wang L, Fett JD, Staniloae C (2012). Predictors of left ventricular recovery in a cohort of peripartum cardiomyopathy patients recruited via the internet. Int J Cardiol.

[40] Felker M, Mann D (2020) A companion to Braunwald's heart disease. Heart failure Vol Fourth Volume Elsevier inc.

[41] Rodriguez Ziccardi M, Siddique MS (2001) Peripartum cardiomyopathy, StatPearls Publishing, 2020 Drugs AAoPCo.Transfer of drugs and other chemicals into human milk.Pediatrics 108:776–89

[42] Halpern DG, Weinberg CR, Pinnelas R, Mehta-Lee S, Economy KE, Valente AM (2019). Use of medication for cardiovascular disease during pregnancy: JACC state-of-the-art review. J Am Coll Cardiol.

[43] Regitz-Zagrosek V, Blomstrom Lundqvist C, Borghi C (2011). ESC Guidelines on the management of cardiovascular diseases during pregnancy: the Task Force on the Management of Cardiovascular Diseases during Pregnancy of the European Society of Cardiology (ESC). Eur Heart J.

[44] Rasmusson K, Brunisholz K, Budge D (2012). Peripartum cardiomyopathy: post-transplant outcomes from the United Network for Organ Sharing Database. J Heart Lung Transplant.

[45] Lindley K, Verma V, Blauwet L (2019). Peripartum cardiomyopathy. Heart Failure Clinics.

[46] Sliwa K, Blauwet L, Tibazarwa K, et al (2010) Evaluation of bromocriptine in the treatment of acute severe peripartum cardiomyopathy: a proof-of-concept pilot study. Circulation 121(13):1465-73. 10.1161/CIRCULATIONAHA.109.901496 10.1161/CIRCULATIONAHA.109.901496 20308616

[47] Yameogo NV, Kagambega LJ, Seghda A, et al (2017) Bromocriptine in management of peripartum cardiomyopathy: A randomized study on 96 women in Burkina Faso. J Cardiol Clin Res 5:1098

[48] Hilfiker-Kleiner D, Haghikia A, Berliner D, et al (2017) Bromocriptine for the treatment of peripartum cardiomyopathy: a multicentre randomized study. Eur Heart J 38(35):2671-2679. 10.1093/eurheartj/ehx355 10.1093/eurheartj/ehx355 PMC5837241 28934837

[49] Mason JW, O'Connell JB, Herskowitz A, et al. (1995) A clinical trial of immunosuppressive therapy for myocarditis The Myocarditis Treatment Trial Investigators N Engl J Med 333(5):269–275 10.1056/NEJM199508033330501 7596370

[50] Drugs AAoPCo (2001) Transfer of drugs and other chemicals into human milk. Pediatrics 108(3):776–789 10.1542/peds.108.3.776 11533352

[51] McNamara DM, Elkayam U, Alharethi R (2015). Clinical outcomes for peripartum cardiomyopathy in North America: results of the IPAC Study (Investigations of Pregnancy- Associated Cardiomyopathy). J Am Coll Cardiol.

[52] Habli M, O'Brien T, Nowack E, Khoury S, Barton JR, Sibai B (2008). Peripartum cardiomyopathy: prognostic factors for long-term maternal outcome. Am J Obstet Gynecol.

[53] Moioli M, Valenzano Menada M, Bentivoglio G, Ferrero S (2010). Peripartum cardiomyopathy. Arch Gynecol Obstet.

[54] Elkayam U, Schäfer A, Chieffo A, et al. (2019) Use of Impella heart pump for management of women with peripartum cardiogenic shock Clin Cardiol 2019;42(10):974–981 (In eng) 10.1002/clc.23249 PMC6788473 31436333

[55] Sieweke JT, Pfeffer TJ, Berliner D, et al. (2020) Cardiogenic shock complicating peripartum cardiomyopathy: importance of early left ventricular unloading and bromocriptine therapy Eur Heart J Acute Cardiovasc Care 9(2):173–182 (In eng) 10.1177/2048872618777876 29792513

[56] Sliwa K, Petrie MC, Hilfiker-Kleiner D, et al. (2018) Long-term prognosis, subsequent pregnancy, contraception and overall management of peripartum cardiomyopathy: practical guidance paper from the Heart Failure Association of the European Society of Cardiology Study Group on Peripartum Cardiomyopathy Eur J Heart Fail 20(6):951–962 (In eng) 10.1002/ejhf.1178 29578284

